# Diagnosis of coexistent neurodegenerative dementias in multiple sclerosis

**DOI:** 10.1093/braincomms/fcac167

**Published:** 2022-06-22

**Authors:** Diana P Londoño, Kogulavadanan Arumaithurai, Eleni Constantopoulos, Michael R Basso, R Ross Reichard, Eoin P Flanagan, B Mark Keegan

**Affiliations:** Department of Neurology, Mayo Clinic, Rochester, MN 55905, USA; Department of Neurology, OSF St. Paul Medical Center, Peoria, IL 61603, USA; Department of Neurology, Mayo Clinic, Rochester, MN 55905, USA; Department of Laboratory Medicine and Pathology, Mayo Clinic, Rochester, MN 55905, USA; Division of Neurocognitive Disorders, Department of Psychiatry and Psychology, Mayo Clinic, Rochester, MN 55905, USA; Department of Laboratory Medicine and Pathology, Mayo Clinic, Rochester, MN 55905, USA; Department of Neurology, Mayo Clinic, Rochester, MN 55905, USA; Department of Neurology, Mayo Clinic, Rochester, MN 55905, USA

**Keywords:** multiple sclerosis, biomarkers, diagnosis, neurodegenerative disease, dementia

## Abstract

Among people with multiple sclerosis, cognitive impairment occurs commonly and is a potent predictor of disability. Some multiple sclerosis patients present with severe cognitive impairment, and distinguishing multiple sclerosis-related cognitive impairment from co-existent progressive neurodegenerative diseases such as Alzheimer disease poses a diagnostic challenge. The use of biomarkers such as PET and CSF proteins may facilitate this distinction. The study was a retrospective, descriptive study on convenience samples of separate cohorts, one of cognitively impaired multiple sclerosis patients evaluated on autopsy to demonstrate coincidence of both multiple sclerosis and neurodegenerative cognitive diseases. The second cohort were cognitively impaired multiple sclerosis patients evaluated by biomarker to investigate possible additional neurodegenerative cognitive disorders contributing to the cognitive impairment. We investigated selected biomarkers among 31 severely impaired patients (biomarker cohort) and 12 severely impaired patients assessed at autopsy and selected 24 (23 biomarker cohort, 1 autopsy cohort) had comprehensive neurocognitive testing. Biomarker cohort investigations included 18F-Fluorodeoxyglucose PET and/or CSF amyloid Aβ1-42, phospho-tau and total tau levels. The autopsy cohort was evaluated with comprehensive neuropathological assessment for aetiology of cognitive impairment. The cohorts shared similar sex, age at multiple sclerosis onset and multiple sclerosis clinical course. The autopsy-cohort patients were older at diagnosis (69.5 versus 57 years, *P* = 0.006), had longer disease duration [median (range) 20 years (3–59) versus 9 (1–32), *P* = 0.001] and had more impaired bedside mental status scores at last follow-up [Kokmen median (range) 23 (1–38) versus 31 (9–34) *P* = 0.01]. Autopsy-cohort patients confirmed, or excluded, coexistent neurogenerative disease by neuropathology gold standard. Most biomarker-cohort patients had informative results evaluating coexistent neurogenerative disease. Biomarkers may be useful in indicating a coexistent neurodegenerative disease earlier, and in life, in patients with multiple sclerosis and significant cognitive impairment.

## Teaser

Londoño *et al.* report some people with multiple sclerosis with significant cognitive impairment have an additional neurodegenerative cognitive disease including Alzheimer changes confirmed at autopsy. Individuals are presented where biomarkers including brain PET and CSF amyloid beta/tau performed in life may indicate such an additional neurodegenerative cognitive disease presence.

## Graphical Abstract

**Graphical abstract:**
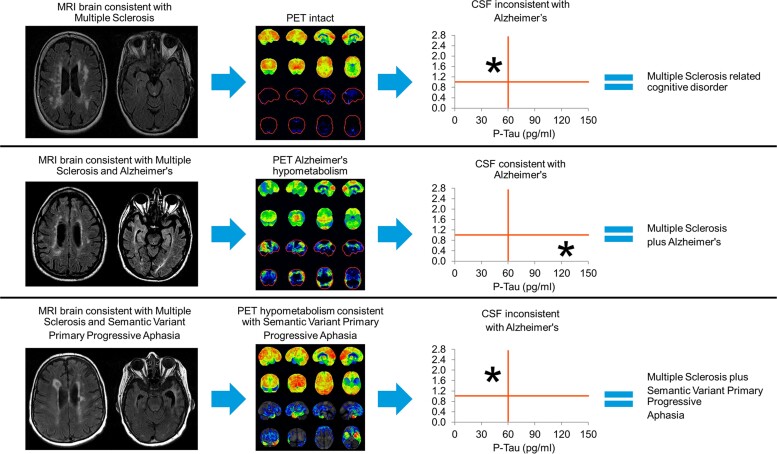

## Introduction

Cognitive dysfunction is an important contributor to disability for people with multiple sclerosis (pwMS). Multiple sclerosis-related cognitive impairment is traditionally described as heterogeneous and mild to moderate in severity.^1,2^ Prevalence studies indicate that 40–60% of pwMS are cognitively impaired, and this can be seen in each stage of the disease.^3,4^ Cognitive impairment often manifests as short-term memory loss, inattention, slowed information-processing speed, executive dysfunction and visuospatial perceptual difficulties.^1,2^ Cognitive phenotypes in pwMS may be distinct with severe phenotypes seen in those with progressive multiple sclerosis.^5^ Severe progressive dementia in relative isolation from other multiple-sclerosis impairment, such as motor weakness, is uncommon but described.^2^ Severe cognitive impairment in pwMS presents a diagnostic and therapeutic dilemma when differentiating multiple sclerosis-associated dementia from multiple sclerosis with an accompanying neurodegenerative dementia.^6^ The importance of identifying accompanying neurodegenerative dementia in life is greatly increasing as revisions to the MS diagnostic criteria have led to earlier diagnosis being possible,^7,8^ late-onset MS is progressively recognized,^9^ MS prevalence in older populations is increasing, ^10^ and increasingly effective symptomatic and disease-modifying therapies become available for neurodegenerative dementias.

CSF biomarkers and imaging (MRI, PET) biomarkers have improved *premortem* diagnosis of neurodegenerative dementias including Alzheimer’s disease, frontotemporal dementia and dementia with Lewy bodies. Small retrospective case series suggested that Alzheimer’s disease biomarkers provide diagnostic clarity, aid prognosis and guide patient counselling and therapeutic decision-making.^11,12^ We aimed to document that pwMS with pronounced cognitive impairment are occasionally confirmed neuropathologically post-mortem to have an accompanying neurodegenerative cognitive disorder. Based on developments in the field of behavioural neurology in diagnosing neurodegenerative dementia with biomarkers premortem, we then aimed to investigate contemporary pwMS with pronounced cognitive impairment who had been assessed for evidence of an accompanying neurodegenerative cognitive disorder using diagnostic biomarkers. We hypothesized that the use of diagnostic biomarkers could suggest the presence of an additional neurodegenerative dementing disease in pwMS previously only discovered at autopsy.

## Materials and methods

### Patients

**Figure 1:**
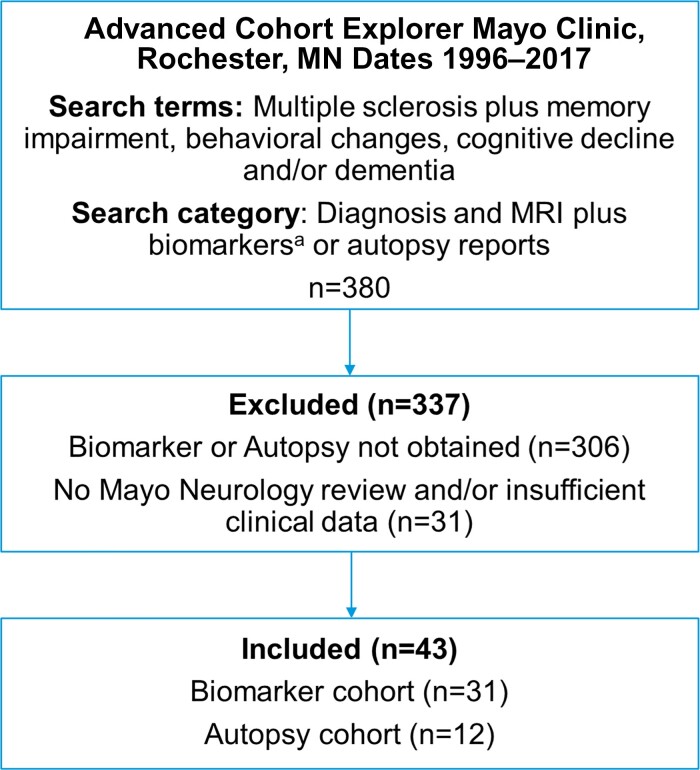
**Patient ascertainment.**  ^a^Biomarkers: FDG-PET, CSF with β-amyloid and tau ratios.

The study was a retrospective, descriptive study on convenience samples of separate cohorts, one with pwMS with cognitive impairment evaluated on autopsy with a goal to demonstrate both MS and neurodegenerative cognitive diseases can occur. The second cohort were pwMS with cognitive impairment evaluated by biomarker evaluations to investigate the possibility of an additional neurodegenerative cognitive disorder potentially contributing to the cognitive impairment. This study included adults (aged 18 years or older) with multiple sclerosis diagnosis undergoing cognitive impairment evaluation at Mayo Clinic, Rochester from 1996 to 2017. The study was approved by the Institutional Review Board of Mayo Clinic, Rochester, Minnesota (IRB 17-010980); subjects provided written consent for the use of their medical information for research purposes. Subjects were identified using the advanced cohort explorer database using the following search terms/diagnostic categories (Fig. 1). Inclusion criteria were as follows: (i) multiple sclerosis diagnosis either in life or pathologically diagnosed demyelinating disease at autopsy consistent with multiple sclerosis^13,14^; (ii) advanced cognitive impairment suspected to be due to either multiple sclerosis in isolation or with suspected or confirmed coexistent neurodegenerative dementia; (iii) biomarker evaluation including 18F-Fluorodeoxyglucose PET (FDG-PET) and/or CSF Alzheimer’s disease biomarkers (amyloid Aβ1–42, phospho-tau and total tau levels); or autopsy evaluation for neurodegenerative dementia. Exclusion criteria were subjects in whom multiple sclerosis was not confirmed with clinical, radiological or pathological evaluation; cognitive status was not evaluated; or in whom the cognitive impairment was directly attributable to an alternative cause (e.g. potentially reversible delirium due to metabolic, nutritional, infectious, or other cause). Electronic medical review was performed for clinical history; neuroimaging was reviewed in all cases. All subjects had brief ‘bedside’ mental status testing.^15,16^ A subgroup of pwMS underwent comprehensive neuropsychological testing. Three patients were reported in a previous study.^11^

### Clinical definitions

Multiple sclerosis was defined by pathologically diagnosed demyelinating disease at autopsy consistent with multiple sclerosis or according to the 2017 revisions to the McDonald Diagnostic Criteria.^17^ The multiple sclerosis course was defined using the 2013 revision of defining clinical course of multiple sclerosis as CIS = clinically isolated syndrome, RRMS = relapsing remitting multiple sclerosis, PPMS = primary progressive multiple sclerosis or SPMS = secondary progressive multiple sclerosis.^18^

Cognitive impairment was defined as significant cognitive impairment suspected to be due to either multiple sclerosis in isolation or with suspected or confirmed coexistent neurodegenerative cognitive impairment as identified through a combination of (i) history taken from the patient or a collateral history from another source; and (ii) an objective cognitive assessment using a bedside mental status examination, data captured from neurologist’s notes during the chart review. Cognitive impairment involved one or more of the following domains: impaired ability to remember new information, impaired reasoning and handling complex tasks or poor judgment, impaired visuospatial abilities, impaired language functions or changes in personality.

We defined the clinical diagnoses as follows: (i) mild cognitive impairment (MCI), defined by cognitive impairment insufficient to fulfil criteria for dementia but more severe than normal aging and was divided into amnestic and non-amnestic subtypes; (ii) probable Alzheimer’s dementia by NIA-AA criteria^19^ with the caveat for the study that an additional active neurological process (multiple sclerosis) may be present; (iii) possible Alzheimer’s disease dementia (insufficient historical detail of progressive decline or atypical clinical course); (iv) behavioural variant frontotemporal dementia (bvFTD) by the International Behavioural Variant Frontotemporal Dementia Criteria Consortium^20^; (v) primary progressive aphasia by clinical criteria ^21^; or (vi) possible ‘multiple sclerosis-exclusive’ cognitive impairment.

### Biomarker

The biomarkers were divided into categories termed amyloid (A), tau (T) and neurodegeneration (N). A refers to the value of a Beta-amyloid biomarker-CSF Ab42; T, the value of a tau biomarker-CSF phospho-Tau; and N, biomarker of neurodegeneration or neuronal injury (FDG-PET, CSF total tau, brain MRI). Biomarkers were described as positive, negative or unavailable. The biomarker-diagnosis core was defined using the A/T/N classification system^22^ as follows: (i) high-likelihood Alzheimer's disease pathology when one positive biomarker on each category was present; (ii) intermediate likelihood Alzheimer's disease when the *T* biomarker was positive in the presence of clinical diagnosis of possible Alzheimer's disease, with negative or unavailable A or N biomarkers; (iii) MCI-intermediate likelihood due to Alzheimer’s pathology when *T* biomarker were positive in the presence of clinical diagnosis of MCI suspected to be associated with Alzheimer’s pathology or Alzheimer’s disease versus multiple sclerosis with negative or unavailable A or N biomarkers; (iv) MCI unlikely to be associated with Alzheimer’s pathology when at least one negative biomarker on each category was presented; (v) probable bvFTD, when clinical criteria for possible bvFTD were met and *N* biomarker was suggestive of bvFTD; (vi) semantic variant primary progressive aphasia (svPPA), when clinical criteria for possible svPPA were met and *N* biomarker was suggestive.^23^

#### Neuroimaging

Mayo Clinic neuroimaging was performed with 1.5 and 3 T MRI Siemens (Munich, Germany) and General Electric (Fairﬁeld, CT) machines. Gadolinium was administered as gadobutrol (Gadovist) 0.1 mmol/kg IV. Imaging was carried out without delay following the Gadovist. FDG-PET scan was performed with low dose, unenhanced, non-diagnostic quality CT images for anatomic co-registration and attenuation correction purposes beginning ∼30 min after radiotracer injection. Neuroradiologic interpretation from diagnostic centres outside of Mayo Clinic directly reviewed on *Q*uick query *R*adiographs and photographs *E*lectronic *A*nalysis and *D*isplay *S*tation platform. Multiple sclerosis lesion burden was stratified as low, mid or high by assessment of a neurologist.

#### Cerebrospinal fluid

CSF evaluation included assessment of cerebrospinal levels of amyloid-β 1–42 peptide, total tau and phospho-tau with ratios utilized to determine if they are consistent with Alzheimer’s disease (Athena diagnostics) in addition to white blood cell count, protein, glucose, oligoclonal bands and immunoglobulin G index.

#### Neuropathology

Neuropathology reports were obtained for patients who underwent post-mortem brain examination. Comprehensive neuropathological assessment was performed following previously described methods for the evaluation of dementia. Alzheimer disease neuropathologic change (ADNC) was defined using most recent NIA-AA consensus criteria.^24^ Three parameters were scored to assess ADNC including (i) beta-amyloid plaque score (Thal stage),^25^ (ii) tau neurofibrillary tangle stage (Braak stage)^26^ and neuritic amyloid plaque score (CERAD).^27^ Using the NIA-AA algorithm, we subsequently translated these results into defined levels of Alzheimer’s disease neuropathologic change: low, intermediate or high, which denote the likelihood of justifying antemortem cognitive impairment. We considered intermediate and high levels of ADNC as adequate justification for antemortem clinical symptoms of cognitive impairment. Low ADNC found in patients with cognitive impairment indicated that alternative pathologies were likely present.

The neuropathological diagnosis of frontotemporal lobar degeneration (FTLD) was made in accordance with suggested subtyping nomenclature,^28^ and Lewy body disease was assessed using the fourth consensus report of the of dementia with Lewy bodies consortium.^29^ Hippocampal sclerosis was defined as the selective loss of neurons and associated gliosis in the CA1 sector and subiculum.^30^ Primary age-related tauopathy (PART) described the presence of tau neurofibrillary tangles in the absence of amyloid plaques, which are commonly observed in medial temporal lobe structures of aged individuals.^31^

#### Neuropsychology

Cognitive functioning, when requested, was assessed using neuropsychological tests completed and performed by multiple providers at Mayo Clinic, Rochester, Minnesota, USA. No uniform battery was administered to all patients. These included a composite mental-status examination, the Dementia Rating Scale-2,^32^ which measures attention, memory, perseveration, visual-construction skill and concept formation. In addition, the Wechsler Adult Intelligence Scale-III measured intellect. Story memory, list learning and visual-design memory were assessed with measures of verbal and visual memory from the Wechsler Memory Scale-III.^33^ In addition, the Auditory Verbal Learning Test was administered to some patients to assess list learning capacity.^34^ The Boston Naming Test assessed confrontation naming skill.^35^ Lexical and semantic fluency were measured with the Controlled Oral Word Association Test from the Multilingual Aphasia Examination.^36^ To assess visual spatial perception, the Judgment of Line Orientation Test^37^ and Complex Figure Copy Test^38^ were administered. The Working Memory Index and Processing Speed Index were administered to assess auditory and visual working memory. Simple speed of visual information processing was indexed by Trail Making Test A.^39^ Regarding executive function, set shifting was measured with Trail Making Test B^39^ and concept formation assessed with the Wisconsin Card Sorting Test.^40^ The Stroop Color Word Test^41^ assessed inhibition. Except for scores on the Wechsler Intelligence and Memory Scales, values were transformed to norm-referenced *z*-scores using the Mayo Older Americans Normative Studies.

### Statistical analysis

Descriptive statistics demonstrated clinical and ancillary testing features using median, ranges or percentages to summarize continuous measures and proportions to summarize categorical variables. Data were analyzed with either the two-tailed *t* test (parametric data) or Fisher exact test (non-parametric data). Neuropsychological evaluation values were corrected for age and education. Impairment was defined as *z*-scores falling at or below the 5^th^ percentile of norm-referenced values, and number of impaired scores summed within cognitive domains and median values are reported.

### Data availability

Anonymized data used for this study are available upon reasonable request from the corresponding author.

## Results

### Demographics and clinical characteristics

The demographics and clinical features of the biomarker and autopsy cohorts are summarized in Table 1.

**Table 1:** Demographic and clinical characteristics

	Biomarker cohort (*n* = 31)	Autopsy cohort (*n* = 12)	P-value
Female *n* (%)	20 (65)	8 (67)	0.95
Age in years, median (range)	57 (37–74)	69.5 (49–88)	0.006
MS onset age in years, median (range)	49 (20–62)	47 (29–68)	0.17
MS duration in years, median (range)	9 (1–32)	20 (3–59)	0.001
MS course, n (%)			0.99
CIS	7 (23)	0	
RRMS	12 (39)	2 (17)	
PPMS	3 (9)	2 (17)	
SPMS	9 (29)	5 (42)	
Unknown^a^	0	3 (24)	
Cognitive symptom onset age in years, median (range)	52 (34–70)	57.5 (48–86)	0.03
Cognitive symptom duration in years, median (range)	2.5 (0–17)	7.5 (0–15)	0.08
Kokmen STMS score (out of 38), median (range)	31 (9–34)	23 (1–38)	0.01

EDSS = Expanded Disability Status Scale; CIS = clinically isolated syndrome, MS = multiple sclerosis, PPMS = primary progressive multiple sclerosis; RRMS = relapsing remitting multiple sclerosis; SPMS = secondary progressive multiple sclerosis.

^a^ Unknown = MS course data were not available in the clinical notes.

#### Biomarker cohort

The patient demographics, multiple sclerosis clinical course and impairment and evaluation of memory impairment of the biomarker cohort are documented in Table 1. Most were women, in middle age, with limited multiple sclerosis-associated gait impairment (Table 1). The cognitive impairment reported symptoms included memory loss and other cognitive domains in 30 of 31 (96%) with one patient presenting solely with behavioural changes.

The evaluations by A/T/N classification and presence and clinical diagnosis of neurodegenerative-dementing disease are reported in Table 2. The most common A/T/N and clinical diagnoses were Alzheimer’s disease and mild cognitive impairment. Most patients had a progressive multiple sclerosis disease course with mild impairment due to multiple sclerosis otherwise and mild multiple sclerosis demyelinating-lesion burden.

**Table 2:** Biomarker cohort: multiple sclerosis patients with cognitive symptoms undergoing biomarker investigation

Case	Gender	Biomarker by A/T/N classification system	Propose diagnosis by A/T/N classification system	Clinical diagnosis	MS course
1	M	A+/T+/N+	High likelihood AD	Probable AD	CIS
2	F	A+/T+/N+	High likelihood AD	Probable AD	RRMS
3	M	A+/T+/N+	High likelihood AD	Probable AD	PPMS
4	F	A+/T+/N+	High likelihood AD	Probable AD	PPMS
5	F	A+/T+/N+	High likelihood AD	Probable AD	CIS
6	F	A+/T+/N+	High likelihood AD	Probable AD	RRMS
7	F	A-/T-/N+	Intermediate likelihood AD	Probable AD	CIS
8	F	Au/Tu/N+	Intermediate likelihood AD	Probable AD	RRMS
9	F	Au/Tu/N+	Intermediate likelihood AD	Probable AD	RRMS
10	F	Au/Tu/N+	Intermediate likelihood AD	Probable AD	PPMS
11	M	Au/Tu/N+	MCI-intermediate likelihood due to AD	MCI due to AD	SPMS
12	F	Au/Tu/N+	MCI-intermediate likelihood due to AD	MCI due to AD	PPMS
13	F	Au/Tu/N+	MCI-intermediate likelihood due to AD	MCI due to AD or MS	SPMS
14	M	Au/Tu/N+	MCI-intermediate likelihood due to AD	MCI due to AD or MS	SPMS
15	F	Au/Tu/N+	MCI-intermediate likelihood due to AD	MCI due to AD or MS	PPMS
16	M	Au/Tu/N+	MCI-intermediate likelihood due to AD	MCI due to AD or MS	CIS
17	M	Au/Tu/N+	MCI-intermediate likelihood due to AD	MCI due to AD or MS	RRMS
18	F	Au/Tu/N-	MCI, unlikely due to AD	MCI due to AD	RRMS
19	F	A-/T-/Nu	MCI, unlikely due to AD	MCI due to AD	SPMS
20	F	A-/T-/N-	MCI, unlikely due to AD	MCI due to AD	SPMS
21	M	A-/T-/N-	MCI, unlikely due to AD	MCI due to AD	CIS
22	M	A-/T-/Nu	MCI, unlikely due to AD	MCI due to AD	RRMS
23	F	A-/T-/Nu	MCI, unlikely due to AD	MCI due to AD	CIS
24	F	A-/T-/N-	MCI, unlikely due to AD	MCI due to AD	CIS
25	F	A-/T-/N-	MCI, unlikely due to AD	MCI due to MS or AD	SPMS
26	F	A-/T-/N-	MCI, unlikely due to AD	MCI due to MS or AD	SPMS
27	M	Au/Tu/N-	MCI, unlikely due to AD	MCI due to FTD	RRMS
28	F	A-/T-/N+	Probable bvFTD	Probable FTD	SPMS
29	F	A-/T-/N+	Probable bvFTD	Probable FTD	SPMS
30	M	A-/T-/N+	Probable bvFTD	Probable FTD	CIS
31	M	Au/Tu/N+	Probable logopenic PPA	Probable PPA	CIS

Biomarker: A refers to the value of an Ab biomarker (CSF Ab42); T, the value of a tau pathology biomarker (CSF p-tau); and N, a quantitative or topographic biomarker of neurodegeneration or neuronal injury (CSF t-tau, FDG-PET, or structural MRI). + = positive; - = negative; u = unavailable. AD = Alzheimer’s dementia; bvFTD = behavioural variant Frontotemporal dementia; PPA = primary progressive aphasia; MCI = mild cognitive impairment; SNAP = suspected non-Alzheimer pathophysiology; CIS = clinical isolated syndrome; RRMS = relapsing remitting multiple sclerosis; PPMS = primary progressive multiple sclerosis; SPMS = secondary progressive multiple sclerosis; MS = multiple sclerosis; EDSS = Expanded Disability Status Scale.

**Figure 2:**
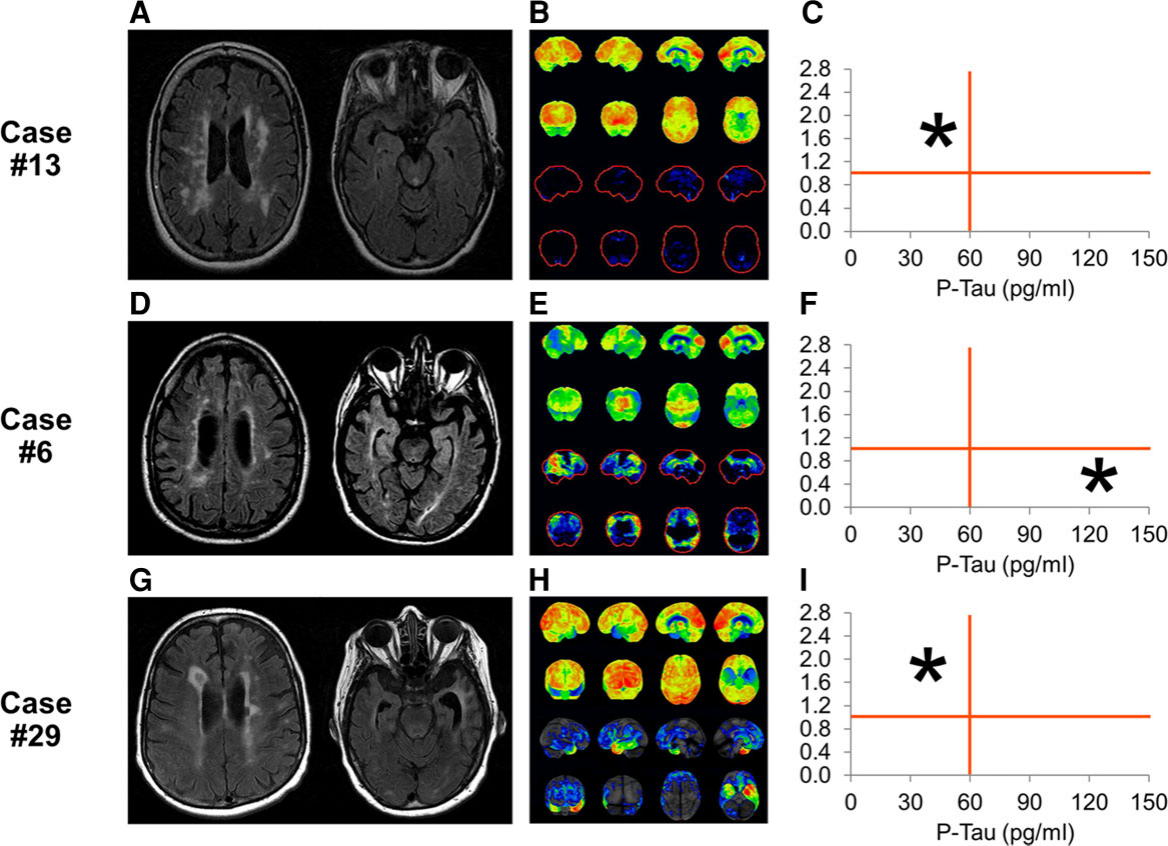
**Illustrative case examples.** MRI of head demonstrating classic periventricular, deep white matter and juxtacortical lesions of multiple sclerosis in all three cases (**A, D and G**). Note prominent hippocampal atrophy bilaterally with AD (**D**) and asymmetric temporal atrophy on the left with svPPA (**G**). FDG-PET (**B, E and H**) demonstrated normal metabolism (**B**), hypometabolism in the posterior cingulate, precuneus, frontal, temporal and parietal lobes bilaterally consistent with AD (**E**) and left more than right anterior, inferior temporal lobe hypometabolism in a patient with svPPA (**H**). CSF AB42, Phospho-Tau and Total tau plotted graph illustrating the position of each patient in relation to cut-off values for not consistent or consistent with AD (**C, F and I**). AD = Alzheimer’s dementia, svPPA = semantic variant primary progressive aphasia.

Representative investigations of brain MRI, PET and CSF in pwMS-related cognitive impairment, multiple sclerosis and Alzheimer’s disease, and multiple sclerosis and behavioural variant frontotemporal dementia are shown in Fig. 2. Brain MRI revealed typical changes of multiple sclerosis with multiple periventricular lesions, juxtacortical and infratentorial T_2_ hyperintense lesions and T_1_ hypointensity. Generalized cerebral atrophy with cerebellar atrophy was common, and two subjects had markedly asymmetrical cerebral atrophy. Brain FDG-PET revealed symmetric FDG cortical activity preserved in parietal, frontal, and temporal lobes in the non-Alzheimer’s disease group. Probable non-Alzheimer’s disease with PET hypometabolism without neurodegenerative dementia pattern was found in 4 subjects. High-likelihood Alzheimer’s disease was found in six subjects and demonstrated by bilateral frontal, parietal and temporal hypometabolism including hypometabolism in the posterior cingulate gyrus. Three patients from the probable non-Alzheimer’s disease group had bilateral frontotemporal lobe hypometabolism consistent with probable bvFTD. CSF evaluation revealed high likelihood of Alzheimer pathology in 6 patients (AT index < 1, 85–94% sensitivity and 83–89% specificity distinguishing Alzheimer’s disease from non-Alzheimer’s disease, phospho-Tau concentration > 61 pg/m). P-tau was lower in the patients with other dementias (bvFTD and PPA).

#### Autopsy cohort

The patient demographics, multiple sclerosis clinical course and impairment, and evaluation of memory impairment of the 12 subjects in the autopsy cohort are documented in Table 1. They resembled the biomarker cohort as most were middle aged women, with significant cognitive impairment and limited multiple sclerosis gait impairment.

The decedents had varying degrees of cognitive impairment or dementia prior to death. No antemortem evaluations of biomarkers were performed for autopsy cases. The subjects’ evaluation at death, their multiple sclerosis clinical course, their cognitive impairment clinical course, and neuropathological findings are summarized in Table 3.

**Table 3:** Evaluation at death, MS clinical course, cognitive impairment course, neuropathology

Case No.	Death (age)	Sex	Neuropathological diagnosis	Clinical diagnosis	Cognitive complaints onset (age in years)	Cognitive complaints duration (age in years)	Bedside mental status^a^	MS symptom onset (age)	MS course
1	56	F	High ADNC	Dementia due to progressive leukoencephalopathy of unknown etiology	50	6	NA	50	PPMS
2	69	F	High ADNC	Probable AD	57	12	1/38	Clinically unrecognized^c^	Unknown^b^
3	70	F	High ADNC	Probable AD	63	7	22/30	47	Unknown
4	49	F	FTLD-MND, chronic infarct	Dementia due to MS	48	1	24/38	34	SPMS
5	66	M	CBD (FTLD-tau)	Probable FTD	55	11	38/38	37	RRMS
6	88	F	HS-TDP-43, PART	MCI due to MS	86	2	22/38	29	SPMS
7	71	M	Low ADNC	MCI due to MS	58	13	NA	58	SPMS
8	65	F	Low ADNC, chronic infarct	MCI due to MS and CVA	65	0	28/38	34	SPMS
9	87	F	PART, chronic infarct	MCI due to MS	74	13	30/30	47	Unknown
10	49	M	ALS	MCI due to MS	48	1	NA	32	RRMS
11	70	M	LBD brainstem-predominant	MCI due to MS and alcohol	62	8	23/38	50	PPMS

^a^ Kokmen mental status where denominator is 38, Mini-mental state exam where denominator is 30.

^b^ Unknown = Data were not available in the clinical notes.

^c^ Clinically unrecognized = MS lesions found during neuropathological examination, data about MS symptoms or MS diagnosis was missing on the clinical notes or was not available.

AD = Alzheimer’s dementia; ADNCs = Alzheimer disease neuropathologic changes; CBD = corticobasal degeneration; FTD = frontotemporal dementia; FTLD-MND = frontotemporal lobar degeneration with motor neuron disease; HS-TDP43 = hippocampal sclerosis (HS) with TAR-DNA binding protein of 43 kDa (TDP-43); LBD = Lewy Body Dementia; MS = multiple sclerosis; PART = primary age-related tauopathy, PPMS = primary progressive multiple sclerosis; RRMS = relapsing remitting multiple sclerosis; SPMS = secondary progressive multiple sclerosis.

In all 12 cases, pathology examination demonstrated multifocal chronic demyelinating plaques of various locations, sizes, and ages, with relative axon preservation. Variable perivascular lymphocytic cuffing, remyelination, and leptomeningeal inflammation were observed. Several cases showed neuropathological changes of multiple sclerosis in the brainstem and spinal cord. Of five individuals with additional Alzheimer’s disease neuropathologic change, three women met criteria for Alzheimer’s disease, and two others showed minimal Alzheimer-type changes. Other neurodegenerative pathologies included FTLD-MND, CBD, hippocampal sclerosis with TDP-43 inclusions (HS), ALS, brainstem-predominant Lewy body disease and PART.

**Figure 3:**
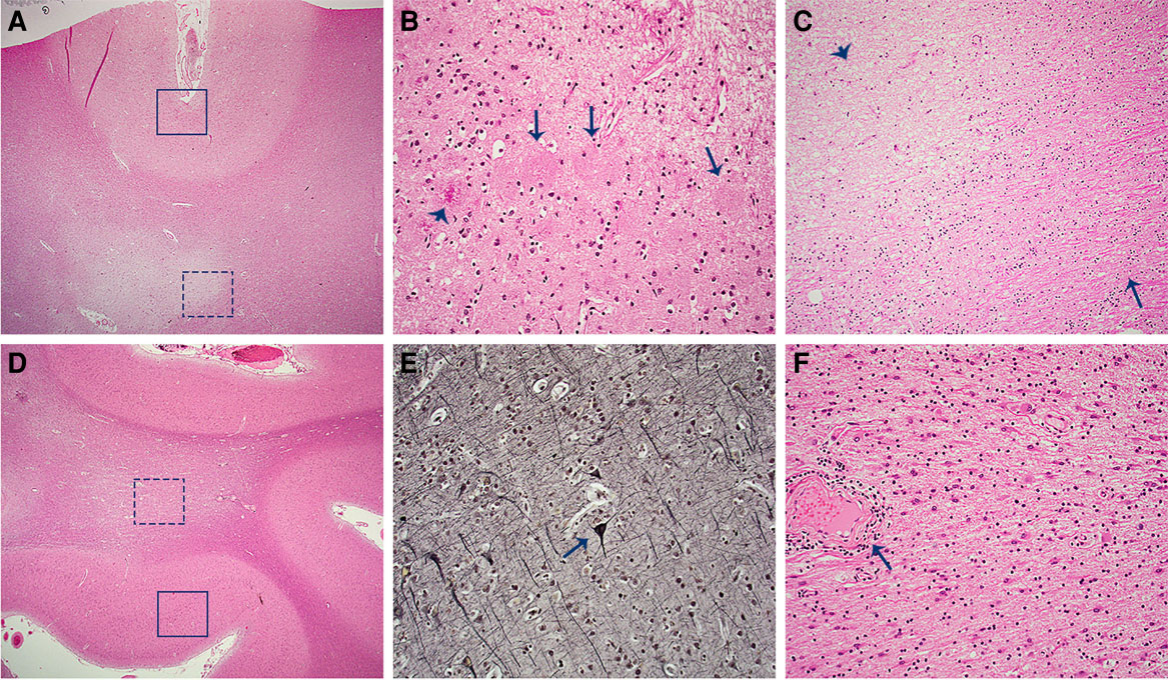
**Cortex and subcortical white matter with concurrent ADNC and lesions of multiple sclerosis in autopsy cohort patient #1.** Frontal lobe (**A**) with cortical amyloid plaques of AD (**B**) including ‘cotton wool plaques’ (arrows) and a dense-cored plaque (arrowhead), and subcortical chronic white matter plaque of multiple sclerosis (**C**) which is well-defined, with hypocellularity and loss of myelin (arrowhead) and preservation of adjacent myelinated axons (arrow). Temporal lobe (**D**) with cortical neurofibrillary tangle of AD (**E**) and subcortical multiple sclerosis plaque (**F**) with scattered reactive astrocytes and perivascular lymphocytic cuffing (arrow). Haematoxylin and eosin (x25) images (**A, D**), haematoxylin and eosin (x200) images (**B, C, F**), Bielschowsky silver (x200) image (**E**).

The three cases with high ADNC were all females with early-onset Alzheimer’s disease (age at death ranged from 56–70) with the onset of cognitive decline averaging 8 years prior to death. For one Alzheimer’s disease case, multiple sclerosis was clinically silent and discovered at autopsy (Fig. 3). The two other women with early-onset Alzheimer’s disease had contrasting multiple-sclerosis courses, with a 23-year history versus a 6-year history of multiple sclerosis.

Among younger patients were a female with FTLD-MND and a man with ALS, each 49 years old. Each developed multiple sclerosis in their early 30 s, and each had a 1-year history of cognitive decline. A 66-year-old man with FTLD-tau, classified as cortical basal syndrome, experienced a 29-year history of multiple sclerosis and 11 years of cognitive impairment. The oldest patient was an 88-year-old woman who developed multiple sclerosis in her 20 s and was cognitively intact until two years prior to death; she was found to have hippocampal sclerosis on post-mortem neuropathological examination.

The following co-pathologies were also observed: cerebral amyloid angiopathy, vascular injury, and cerebrovascular disease. Within the autopsy cohort, three cases had remote infarcts (cases 4, 8 and 9). In each case, a single chronic infarct was identified, two were within cortical regions and one within the striatum. Although cognitive impairment can develop following a single ischaemic lesion, it is more often associated with small, widespread ischaemic lesions and less commonly associated with a focal infarct.^42^ Cognitive impairment due to a focal vascular lesion is typically associated with lesions strategically located in functionally significant areas. The cortical infarcts in Cases 8 and 9 involved the frontal lobe and the parietal lobe, respectively, outside of known, functionally significant cortical areas associated with vascular cognitive impairment.^42^ These were clinically silent and incidentally noted on later imaging as areas of encephalomalacia. In case 4, the striate nucleus infarct was superimposed on an MS plaque and was unclear on imaging. This lesion was clinically diagnosed and led to motor impairment; no change in cognition following the event was noted. Of all three cases of ischaemic lesions, no clinical signs of post-stroke cognitive impairment, a stepwise progression of cognitive decline following a diagnosed stroke, were noted.^43^ All cases had mild-to-moderate arteriolosclerosis and negligible intracranial atherosclerosis.

### Neuropsychometric testing

Comprehensive neuropsychological testing was performed in 24 patients (23, biomarker group; 1, autopsy cohort). The mean (range) age was 59 (46–74), and 15 (63%) were female. The median (range) education was 15 years (12–19). While no uniform battery was administered to all patients, the details of the neuropsychological testing are presented in Supplementary Table 1. There were no clear discriminating features to assess multiple sclerosis-related cognitive impairment in isolation from coexistent neurodegenerative-dementing diseases.

## Discussion

This study suggests that further investigations with in vivo biomarkers and confirmation with gold standard neuropathological assessment are indicated to attempt to discover coexistent neurodegenerative cognitive disorders in pwMS and prominent cognitive impairment.’ The cause of a coexistent neurodegenerative cognitive disorder may, in some cases, now be suggested strongly in life by appropriate investigative biomarkers. This discovery, if confirmed, may allow pwMS to be directed to appropriate, currently approved symptomatic and disease-modifying therapies for neurodegenerative cognitive disorders.

The diagnostic and therapeutic dilemma differentiating cognitive impairment due to multiple sclerosis from that with coexistent neurodegenerative cognitive disorders will likely become more common as multiple sclerosis is diagnosed later in life^10,44^ with successful multiple sclerosis disease-modifying therapies prolonging life^45^ further into the elderly age range in which degenerative dementias more commonly occur. Treatment of neurodegenerative dementia has remained challenging^46,47^; however, increasingly approved symptomatic (e.g. acetylcholinesterase inhibitors, memantine) and potentially disease-modifying therapies (e.g. aducanumab, donanemab) are on the horizon.

One barrier to diagnosing coexisting neurodegenerative disorders in pwMS is that the gold standard to diagnose neurodegenerative disorders is still neuropathologic findings on autopsy. This highlights the importance of continued efforts in discovery and refinement of reliable serological, CSF, and imaging biomarkers of neurodegenerative cognitive disorders.

Comprehensive neuropsychology testing may refine bedside suspicion of cognitive impairment due solely to multiple sclerosis or in combination with an additional neurodegenerative cause. Cognitive dysfunction presents differently in multiple sclerosis and Alzheimer’s disease. Classical Alzheimer’s disease has been characterized as cortical dementia.^48^ Multiple sclerosis is often considered a subcortical dementia^49^; however, cortical grey matter is affected by multiple sclerosis.^50–52^ In our study, comprehensive neuropsychometric evaluation showed gross cognitive dysfunction across most of the domains assessed, making it difficult to differentiate on that metric alone between those suspected either to have, or not to have, coexistent neurodegenerative cognitive disorders in addition to MS.

Biomarkers are becoming increasingly important in distinguishing different dementias.^53–55^ Using the A/T/N classification scheme for Alzheimer’s disease biomarkers, we were able to propose that biomarkers distinguish coexistent neurodegenerative dementia subtypes in pwMS as contributors to cognitive impairment. The literature concerning comorbid multiple sclerosis and neurodegenerative illness is limited, and incidence and prevalence of comorbidity are lacking. In a review of comorbid multiple sclerosis and Alzheimer’s disease, Luczynski *et al*.^56^ identified six case studies and case-series reports of cognitively impaired elderly patients with multiple sclerosis who progressively demented. Largely through post-mortem methods, they were later determined to have both multiple sclerosis and Alzheimer’s disease. Luczynski *et al*. concluded that efforts to identify antemortem markers should be pursued. Subsequently, Jakimovski *et al*.^57^ reported a case of an 84-year old patient with multiple sclerosis who manifested salient cognitive impairment. Over 13 years, the patient was assessed repeatedly, and amyloid-based PET imaging and neurocognitive testing revealed an evolving presentation that indicated amnestic mild cognitive impairment. The authors concluded that antemortem methods can identify pwMS who develop cognitive impairment associated with multiple sclerosis and neurodegenerative conditions. Further tau imaging and new biomarkers of neurodegenerative disease of multiple sclerosis cases with early and severe cognitive decline will be useful to confirm these results and better appreciate the frequency of coexisting dementing pathologies.^58^

This study has limitations, as it was a highly selected, convenience sample at an academic institution with small sample size. Clinical assessment, comprehensive neuropsychological assessment, neuroimaging, CSF biomarkers, and neuropathology were done inconsistently and only directed by routine clinical care. We do not know with certainty how those with biomarker investigations or autopsied pwMS compared with the overall population of those pwMS with similar clinical presentations who, for reasons of provider and patient experience, expectations and many other undetermined issues, were not investigated with biomarkers or presented to autopsy. Presumably, people with MS in whom a comorbid neurodegenerative condition was suspected on clinical grounds (e.g. severity of neurocognitive symptoms and signs) were more likely to be selected for such evaluations. To demonstrate definitively that biomarkers improve diagnostic accuracy of an accompanying neurodegenerative cognitive disease in people with MS would require a more rigorous evaluation of pwMS and cognitive impairment with a standard set of biomarker evaluations (including brain PET and CSF amyloid and tau) and follow-up each to neuropathological autopsy evaluation. Despite this, informative data on this increasingly common clinical situation were gained, and further prospective, controlled studies incorporating neurodegenerative biomarkers and subsequent neuropathological assessment are indicated.

While we cannot entirely exclude some contribution of the neurodegenerative changes of MS itself or of additional cerebrovascular disease, brain PET biomarker has been shown to discriminate Alzheimer's disease from cognitively impaired controls (including MCI) with 92% sensitivity (95% CI: 84–96%) and 78% specificity (95% CI: 69–85%) PMID: 21694448.^59^ While some changes in biomarkers are reported in MS, such as global and regional hypometabolism on FDG-PET ^60^ and reduced CSF amyloid beta in CSF,^61^ the PET hypometabolism patterns in our patients were suggestive of an additional neurodegenerative dementing cause. CSF tau is markedly elevated in Alzheimer's disease, but reports in MS are variable with mild elevations noted at initiation of demyelinating disease and similar values to controls in others with established MS.^62^ Although the diagnosis of probable Alzheimer's disease requires exclusion of another concurrent active neurological disease and MS diagnostic criteria also insist that there must be ‘no better explanation,’ the challenge of this study was to document pathological confirmation of MS and an additional neurodegenerative cognitive disorder and then assess the use of contemporary biomarkers to explain the clinical presentation of MS *and* an additional neurodegenerative cognitive disorder.

FDG-PET of vascular dementia differs in its pattern of hypometabolism when compared to other neurodegenerative dementia (e.g. vascular dementia: focal cortical, subcortical, deep grey nuclei and cerebellar hypometabolism versus Alzheimer's disease: posterior cingulate cortex, precuneus and parietotemporal to frontal cortex hypometabolism).^63^ CSF biomarkers for vascular dementia differ from Alzheimer's disease and in our study the findings were more suggestive of AD.^64^

Our study contributes to the existing knowledge about the use of biomarkers of neurodegenerative dementing diseases in the setting of cognitive impairment in multiple sclerosis.

## Supplementary Material

fcac167_Supplementary_Data

## Abbreviations

A =: amyloid
ADNC =: Alzheimer disease neuropathologic change
bvFTD =: behavioural variant frontotemporal dementia
CERAD =: Consortium to Establish a Registry for Alzheimer's Disease
CIS =: Clinically isolated syndrome
FDG =: 18F-Fluorodeoxyglucose
FTLD =: frontotemporal lobar degeneration
MCI =: mild cognitive impairment
N =: neurodegeneration
NIA-AA =: National Institute on Aging-Alzheimer’s Association
PART =: primary age-related tauopathy
PPMS =: primary progressive multiple sclerosis
pwMS =: people with multiple sclerosis
RRMS =: relapsing remitting multiple sclerosis
SPMS =: secondary progressive multiple sclerosis
svPPA =: semantic variant primary progressive aphasia
T =: tau
