# Supplementary material for: Diagnosis of coexistent neurodegenerative dementias in multiple sclerosis
Source: Brain Commun. 2022 Jun 22;4(4):fcac167. doi: 10.1093/braincomms/fcac167 (PMC9272064; doi:10.1093/braincomms/fcac167)
Supplement: fcac167_Supplementary_Data [file fcac167_supplementary_data.pdf]

Supplementary Material for Diagnosis of Coexistent Neurodegenerative Dementias in Multiple Sclerosis

by Londoño et al.

Contents

Neuropsychological Assessment (text)

Supplementary Table 1

Supplementary Table 2 (Unabridged version of Table 2)

Neuro psychological assessment (Supplementary)

On the Dementia Rating Scale-2, 75% of those tested demonstrated impaired scores, suggesting that gross cognitive dysfunction was present among most of the patients. Notably, this impairment manifested in nearly half of the patients across most of the domains assessed. Approximately 40% of the sample showed Full Scale IQ scores that fell at the 5th percentile, and another 10% of the sample achieved scores that fell at the 16th percentile for their age. This is remarkable, because most of the patients had college or graduate education, suggesting that significant declines in intellect had occurred.

Regarding memory function, nearly half of the sample demonstrated impaired immediate recall of a word list, short stories, and visual designs. Delayed recall of short stories and visual designs was less often impaired, but 60% of the sample demonstrated impaired ability to retain items from word lists.

Confrontation naming was impaired in nearly 25% of the sample. Lexical and semantic fluencies were impaired in approximately 30% of the sample. Thus, language function seemed to be less often compromised than memory.

Visuospatial perception was compromised in nearly 40% of the sample, as was visual working memory. Speed of visual information processing was impaired in nearly a third of the sample. In contrast, auditory working memory was impaired in 21% of the sample.

Executive dysfunction was particularly compromised in the sample, with half of those tested demonstrating impaired capacity for set shifting and inhibition. Concept formation was less commonly impaired and was deficient in approximately one fourth of those tested.

Employing a conservative threshold to define meaningful impairment, as many as 50% of this clinically referred sample displayed impaired executive function, and 40 to 50% of these individuals showed impaired memory, working memory, and visuospatial perception and construction. Language skills were less vulnerable but were not without impairment. Classic studies of Rao[1]revealed that the most common neuropsychological impairments in MS involved executive function, new learning, working memory, and visuospatial perception. In contrast to those earlier studies, intellect typically was not compromised except among those patients who displayed severe impairment. Additionally, the frequency of impairment in the current study is somewhat more apparent than in previous prevalence studies of neurocognitive deficits in MS.

Supplementary table 1. Neuropsychological Performance						
	N	Z-Score, median (range)	Normal	Mildly Low	Impaired	Percent Impaired (%)
**Mental Status Examination**						
Dementia Rating Scale-2 Total Score	12	-2.0 (-2.67 to -.33)	1	2	9	75%
**Intellect**						
Full Scale IQ	19	-1.00 (-2.33 to 1.33)	10	2	7	37%
Verbal Comprehension Index	19	-0.50 (-3.00 to 1.67)	11	2	3	16%
Perceptual Organization Index	19	-0.75 (-2.00 to 0.75)	11	3	5	26%
**Learning & Memory**						
Verbal List Learning	15	-1.33 (-2.67 to 0.33)	7	1	7	47%
List Delayed Recall	15	-1.67 (-2.67 to 0.0)	4	2	9	60%
Story Learning	18	-1.0 (-2.67 to 1.67)	9	1	8	44%
Story Learning Delayed Recall	18	-1.0 (-3.0 to 1.0)	9	2	7	39%
Visual Learning	18	-1.33 (-3.0 to 1.0)	8	2	8	44%

Visual Delayed Recall	18	-0.67 (-2.67 to 1.33)	12	3	3	17%
**Language**						
Confrontation Naming	13	-0.67 (-2.67 to 1.0)	8	2	3	23%
Lexical Fluency	21	-0.67 (-3.0 to 1.67)	12	3	6	29%
Semantic Fluency	19	-1.33 (-3.0 to 0.67)	8	4	7	37%
**Visuospatial**						
Visuospatial Perception	19	-.67 (-2.67 to 1.33)	11	1	7	37%
**Attention**						
Auditory Working Memory	14	-.67 (-2.5 to 1.75)	7	4	3	21%
Visual Attention	15	0.75 (-2.67 to 0.33)	5	4	6	40%
Visual Scanning Speed	22	-1.00 (-3.00 to 1.00)	13	2	7	32%
**Executive Function**						
Set-Shifting	22	-2.67 (-3.0 to 0.67)	8	2	11	52%
Concept Formation	9	-0.59 (-1.87 to 1.12)	5	2	2	22%
Inhibition	12	-1.33 (-3.0 to 1.0)	4	2	6	50%

Notes: Tests used for each domain: Mental Status Examination=Dementia Rating Scale-2 Composite Score; Intelligence = Wechsler Adult Intelligence Scale-III Full Scale IQ, Verbal Comprehension Index, Perceptual Organization Index; Verbal List Learning and List Delayed Recall = Auditory Verbal Learning Test or Serial List Learning (from Wechsler Memory Scale-III); Visual Learning and Visual Delayed Recall=Wechsler Memory Scale – III Visual Reproduction Immediate and Delayed Recalls; Confrontation Naming = Boston Naming Test;

Lexical Fluency = Controlled Oral Word Association Test; Semantic Fluency = Category Fluency; Visuospatial Perception= Judgment of Line Orientation Test or Rey Complex Figure Copy Score; Auditory Working Memory = Wechsler Adult Intelligence Scales – III Working Memory Index; Visual Attention = Wechsler Adult Intelligence Scales – III Processing Speed Index; Visual Scanning Speed = Trail Making Test – A; Set Shifting = Trail Making Test – B; Concept Formation= Wisconsin Card Sorting Test Perseverative Errors; Inhibition=Stroop Color Word Test.

Test scores were transformed to norm referenced z-scores. Within Normal Limits = No. of patients whose performance fell no lower than one standard deviation below the normative mean (i.e., no lower than 16th percentile). Mildly Low = No. of patients whose performance fell no lower than 1.5 standard deviations below the normative mean (i.e., 15th to 6th percentile). Impaired = No. of patients whose performance fell below 1.5 standard deviations below the normative mean (i.e., 5th percentile and lower).

Suppl. Table 2. Biomarker Cohort: MS patients with cognitive symptoms undergoing biomarker investigation

Case	Gender	Biomarker by A/T/N classification system	Propose diagnosis by A/T/N classification system	Clinical diagnosis	MS course
1	M	A+/T+/N+	High likelihood AD	Probable AD	CIS
2	F	A+/T+/N+	High likelihood AD	Probable AD	RRMS
3	M	A+/T+/N+	High likelihood AD	Probable AD	PPMS
4	F	A+/T+/N+	High likelihood AD	Probable AD	PPMS
5	F	A+/T+/N+	High likelihood AD	Probable AD	CIS
6	F	A+/T+/N+	High likelihood AD	Probable AD	RRMS
7	F	A-/T-/N+	Intermediate likelihood AD	Probable AD	CIS
8	F	Au/Tu/N+	Intermediate likelihood AD	Probable AD	RRMS
9	F	Au/Tu/N+	Intermediate likelihood AD	Probable AD	RRMS
10	F	Au/Tu/N+	Intermediate likelihood AD	Probable AD	PPMS
11	M	Au/Tu/N+	MCI-intermediate likelihood due to AD	MCI due to AD	SPMS
12	F	Au/Tu/N+	MCI-intermediate likelihood due to AD	MCI due to AD	PPMS
13	F	Au/Tu/N+	MCI-intermediate likelihood due to AD	MCI due to AD or MS	SPMS
14	M	Au/Tu/N+	MCI-intermediate likelihood due to AD	MCI due to AD or MS	SPMS
15	F	Au/Tu/N+	MCI-intermediate likelihood due to AD	MCI due to AD or MS	PPMS
16	M	Au/Tu/N+	MCI-intermediate likelihood due to AD	MCI due to AD or MS	CIS
17	M	Au/Tu/N+	MCI-intermediate likelihood due to AD	MCI due to AD or MS	RRMS
18	F	Au/Tu/N-	MCI, unlikely due to AD	MCI due to AD	RRMS
19	F	A-/T-/Nu	MCI, unlikely due to AD	MCI due to AD	SPMS
20	F	A-/T-/N-	MCI, unlikely due to AD	MCI due to AD	SPMS
21	M	A-/T-/N-	MCI, unlikely due to AD	MCI due to AD	CIS
22	M	A-/T-/Nu	MCI, unlikely due to AD	MCI due to AD	RRMS
23	F	A-/T-/Nu	MCI, unlikely due to AD	MCI due to AD	CIS
24	F	A-/T-/N-	MCI, unlikely due to AD	MCI due to AD	CIS
25	F	A-/T-/N-	MCI, unlikely due to AD	MCI due to MS or AD	SPMS
26	F	A-/T-/N-	MCI, unlikely due to AD	MCI due to MS or AD	SPMS
27	M	Au/Tu/N-	MCI, unlikely due to AD	MCI due to FTD	RRMS
28	F	A-/T-/N+	Probable bvFTD	Probable FTD	SPMS
29	F	A-/T-/N+	Probable bvFTD	Probable FTD	SPMS
30	M	A-/T-/N+	Probable bvFTD	Probable FTD	CIS
31	M	Au/Tu/N+	Probable logopenic PPA	Probable PPA	CIS

Abbreviations: Biomarker: A refers to the value of an Ab biomarker (CSF Ab42); T, the value of a tau pathology biomarker (CSF p-tau); and N, a quantitative or topographic biomarker of neurodegeneration or neuronal injury (CSF t-tau, FDG-PET, or structural MRI). + = positive; - = negative; u = unavailable. AD= Alzheimer's dementia, bvFTD= behavioral variant Frontotemporal dementia, PPA= Primary Progressive Aphasia. MIC= Mild Cognitive Impairment, SNAP= Suspected non-Alzheimer pathophysiology, CIS= Clinical Isolated Syndrome, RRMS= Relapsing Remitting Multiple Sclerosis, PPMS= Primary Progressive Multiple Sclerosis, SPMS= Secondary Progressive Multiple Sclerosis, MS= Multiple Sclerosis. EDSS=**Expanded Disability Status Scale**
